# JAZF1-SUZ12 dysregulates PRC2 function and gene expression during cell differentiation

**DOI:** 10.1016/j.celrep.2022.110889

**Published:** 2022-05-31

**Authors:** Manuel Tavares, Garima Khandelwal, Joanne Muter, Keijo Viiri, Manuel Beltran, Jan J. Brosens, Richard G. Jenner

**Affiliations:** 1 UCL Cancer Institute and Cancer Research UK UCL Centre, University College London (UCL), London WC1E 6BT, UK; 2 Warwick Medical School, Division of Biomedical Sciences, University of Warwick, Coventry CV4 7AL, UK

**Keywords:** chromatin, epigenetics, polycomb, histone modification, endometrium, endometrial stromal sarcoma, embryonic stem cell, cell differentiation, decidualization, histone acetylation

## Abstract

Polycomb repressive complex 2 (PRC2) methylates histone H3 lysine 27 (H3K27me3) to maintain gene repression and is essential for cell differentiation. In low-grade endometrial stromal sarcoma (LG-ESS), the PRC2 subunit SUZ12 is often fused with the NuA4/TIP60 subunit JAZF1. We show that JAZF1-SUZ12 dysregulates PRC2 composition, genome occupancy, histone modification, gene expression, and cell differentiation. Loss of the SUZ12 N terminus in the fusion protein abrogates interaction with specific PRC2 accessory factors, reduces occupancy at PRC2 target genes, and diminishes H3K27me3. Fusion to JAZF1 increases H4Kac at PRC2 target genes and triggers recruitment to JAZF1 binding sites during cell differentiation. In human endometrial stromal cells, JAZF1-SUZ12 upregulated PRC2 target genes normally activated during decidualization while repressing genes associated with immune clearance, and JAZF1-SUZ12-induced genes were also overexpressed in LG-ESS. These results reveal defects in chromatin regulation, gene expression, and cell differentiation caused by JAZF1-SUZ12 that may underlie its role in oncogenesis.

## Highlights

• JAZF1-SUZ12 fails to bind PRC2 subunits due to loss of the SUZ12 N terminus
• JAZF1-SUZ12 switches to a JAZF1-like binding profile during cell differentiation
• JAZF1-SUZ12 reduces H3K27me3 and increases H4Kac at polycomb target genes
• JAZF1-SUZ12 activates polycomb target genes and dysregulates hEnSC decidualization

## Graphical abstract

**Figure:**
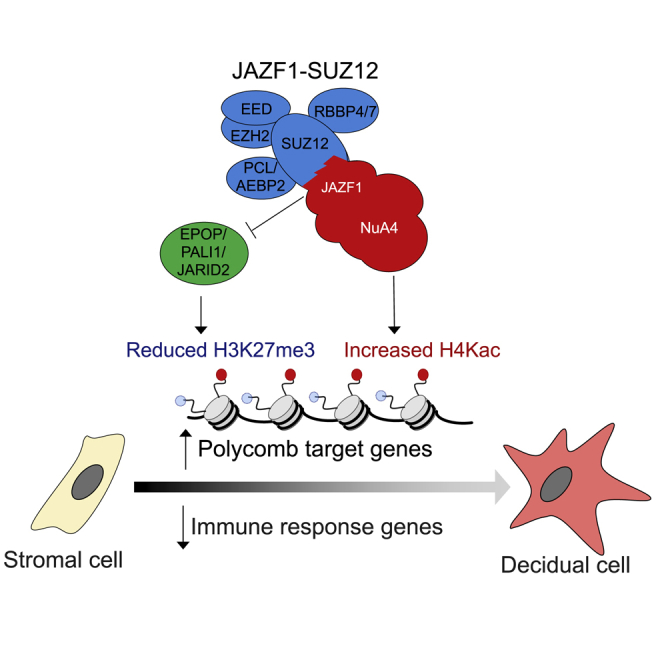

## Teaser

Fusion of JAZF to SUZ12 often occurs in endometrial stromal sarcoma, but the effects of JAZF1-SUZ12 on chromatin and cell differentiation are not understood. Tavares et al. demonstrate that JAZF1-SUZ12 alters PRC2 composition and genome occupancy, reduces H3K27me3 and increases H4Kac, and dysregulates gene expression and endometrial stromal cell decidualization.

## Introduction

Human endometrial stromal cells (hEnSCs) form part of the uterine mucosa, and their differentiation (decidualization) during each menstrual cycle drives menstrual shedding followed by tissue repair and rapid proliferation. Endometrial stromal-cell tumors are divided into four subtypes: benign endometrial stromal nodules (ESNs), low-grade endometrial stromal sarcoma (LG-ESS), high-grade endometrial stromal sarcoma, and undifferentiated uterine sarcoma. Around 50% of ESN and LG-ESS cases exhibit the chromosomal rearrangement t(7; 17) (p15:q21) (Chiang et al., 2011; Micci et al., 2016), which results in production of a JAZF1-SUZ12 fusion protein comprising the first 128 amino acids of JAZF1 in place of the first 93 amino acids of SUZ12 (Koontz et al., 2001). The wild-type (WT) SUZ12 allele remains active in ESN but is silenced in LG-ESS, suggesting this repression is important for tumorigenesis (Li et al., 2007). Forty percent of LG-ESS patients do not respond to current therapeutic regimens (Amant et al., 2007; Beck et al., 2012), highlighting the need for greater understanding of JAZF1-SUZ12 function.

SUZ12 is a core subunit of PRC2, along with EZH2 or EZH1, EED, and RBBP4 or RBBP7 (Margueron and Reinberg, 2011). PRC2 associates with genes encoding developmental regulators specific for other cell types or differentiation stages (Azuara et al., 2006; Boyer et al., 2006; Bracken et al., 2006; Lee et al., 2006). At these genes, PRC2 trimethylates histone H3 lysine 27 (H3K27me3), which allows binding of the canonical form of PRC1 and formation of a repressive chromatin structure. Mice lacking *Suz12* die during embryogenesis due to defects in gastrulation, and this role for SUZ12 in early development is reflected by its requirement for mouse embryonic stem cell (ESC) differentiation *in vitro* (Lee et al., 2006; Pasini et al., 2004; Pasini et al., 2007). PRC2 is also required for the differentiation of numerous other cell types, including endometrial stromal cells (Grimaldi et al., 2011; Nancy et al., 2018; Osokine et al., 2022).

In addition to the core PRC2 subunits, accessory factors define two PRC2 variants: PRC2.1, which contains PCL1, PCL2, or PCL3 (also named PHF1, MTF2, and PHF19) along with either EPOP, PALI1, or PALI2, and PRC2.2, containing AEBP2 and JARID2 (Alekseyenko et al., 2014; Beringer et al., 2016; Conway et al., 2018; Grijzenhout et al., 2016; Hauri et al., 2016; Liefke et al., 2016; Zhang et al., 2011). The accessory subunits act in combination to recruit PRC2 to its target sites (Højfeldt et al., 2018, 2019; Oksuz et al., 2018; Perino et al., 2018; Wang et al., 2017; Youmans et al., 2018, 2021) and are essential for development (Brien et al., 2012; Conway et al., 2018; Grijzenhout et al., 2016; Landeira et al., 2010; Li et al., 2010, 2017; Liefke et al., 2016; Shen et al., 2009; Walker et al., 2010; Zhang et al., 2011). SUZ12 interacts with EZH2 and EED through its C-terminal VEFS domain and with JARID2, AEBP2, EPOP, and PCL1–3 through other parts of the protein (Chen et al., 2018; Kasinath et al., 2018; Kloet et al., 2016; Youmans et al., 2018).

The effect of JAZF1-SUZ12 on PRC2 function is not well understood. Ectopic expression of JAZF1-SUZ12 and knockdown of endogenous SUZ12 in HEK293 cells increased cell proliferation and resistance to hypoxia (Li et al., 2007). JAZF1 fusion has been reported to reduce interaction of SUZ12 with EZH2 and EED (Ma et al., 2016) and prevent interaction with JARID2 and EPOP (Chen et al., 2018), but its effects on other PRC2 accessory factors have not been measured.

Compared with SUZ12, understanding of JAZF1 function is more limited. JAZF1 interacts with the orphan nuclear receptor NR2C2 (TAK1/TR4), inhibiting its activity (Nakajima et al., 2004), and regulates genes with functions in translation and splicing (Kobiita et al., 2020; Procida et al., 2021). Shedding more light on the mechanism of JAZF1 action, the protein was found to be associated with the NuA4/TIP60 histone acetyltransferase complex (Piunti et al., 2019; Procida et al., 2021), which exchanges H2A for H2A.Z (Gévry et al., 2007; Pradhan et al., 2016; Xu et al., 2012) and acetylates histone H4 (H4Kac), as well as histone H2A and its variants (Steunou et al., 2014). Correspondingly, fusion of JAZF1 and SUZ12 results in ectopic interaction between PRC2 and NuA4 components (Piunti et al., 2019), which is consistent with reports of other fusion events between PRC2 and NuA4 subunits in LG-ESS (Micci et al., 2006; Panagopoulos et al., 2008, 2012; Dewaele et al., 2014; Davidson and Micci, 2017; Makise et al., 2019; Han et al., 2020) and indicates that PRC2-NuA4 fusion drives oncogenesis in all these cases. However, how JAZF1-SUZ12 affects PRC2 composition, genome occupancy, and histone modification and the impact of these changes on gene expression and cell differentiation remain unclear.

Here, we show that JAZF1-SUZ12 disrupts interaction of PRC2 with JARID2, EPOP, and PALI1; alters PRC2 genome occupancy; and fails to establish repressive chromatin at polycomb target sites. Consistent with this, JAZF1-SUZ12 ectopically activates polycomb target genes in hEnSCs, and these genes are also upregulated in LG-ESS. We propose that these effects contribute to JAZF1-SUZ12-driven oncogenesis.

## Results

### JAZF1-SUZ12 lacks interaction with JARID2, EPOP, and PALI1 due to loss of the SUZ12 N terminus

**Figure 1:**
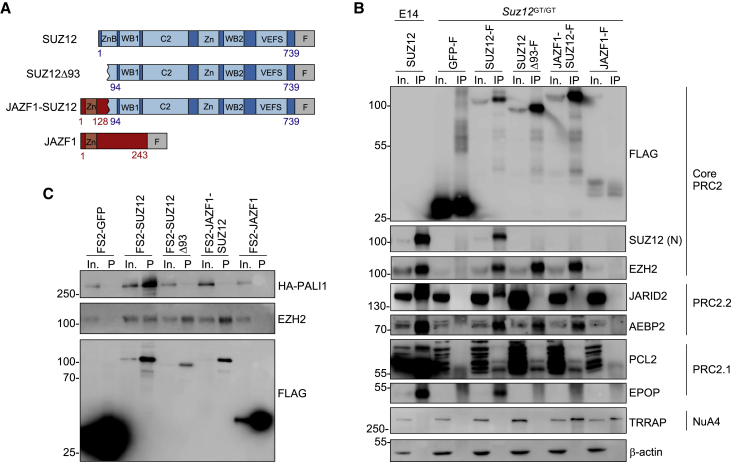
JAZF1-SUZ12 lacks interaction with JARID2, EPOP, and PALI1 due to loss of the SUZ12 N terminus (A) Models of FLAG-tagged (F) SUZ12, SUZ12Δ93, JAZF1-SUZ12, and JAZF1 constructs. (B) Immunoblots for FLAG, SUZ12 (N terminus), EZH2, JARID2, AEBP2, PCL2, EPOP, TRRAP, and β-actin in input (In.) and SUZ12 IPs from WT or *Suz12*^GT/GT^ ESCs stably expressing FLAG-tagged GFP, SUZ12, SUZ12Δ93, JAZF1-SUZ12, or JAZF1. Data are representative of two independent IPs. (C) Immunoblots for FLAG, HA-PALI, and EZH2 in input and Strep-Tactin pull-downs (P) from NIH3T3 cells transfected with HA-PALI1 and FS2-tagged GFP, SUZ12, SUZ12Δ93, JAZF1-SUZ12, or JAZF1. Data are representative of two independent pull-downs. See also Figure S1.

We first sought to establish the effect of JAZF1-SUZ12 on PRC2 composition. To address this, we generated *Suz12*^GT/GT^ ESC lines stably expressing FLAG-tagged SUZ12, JAZF1-SUZ12, JAZF1, or GFP (Figure 1A). To distinguish between effects caused by loss of the SUZ12 N terminus versus gain of the JAZF1 N terminus, we also generated a cell line stably expressing FLAG-tagged SUZ12Δ93. Immunoblotting confirmed that the levels of SUZ12, SUZ12Δ93, and JAZF1-SUZ12 were similar to each other and to endogenous SUZ12, while JAZF1 was expressed at a lower level (Figure S1A).

Co-immunoprecipitation revealed that SUZ12Δ93 and JAZF1-SUZ12 interacted with EZH2 and the accessory subunits AEBP2 and PCL2, but not with JARID2 or EPOP (Figure 1B). These findings were confirmed by reciprocal co-immunoprecipitations (coIPs) (Figures S1B–S1D). To determine the effect of JAZF1-SUZ12 on the association of PRC2 with PALI1, we co-transfected FS2-tagged SUZ12, SUZ12Δ93, JAZF1-SUZ12, and JAZF1 with hemagglutinin (HA)/FLAG-tagged PALI1 and performed Strep-Tactin affinity purification (Figure 1C). We found that PALI1 did not interact with SUZ12Δ93 or JAZF1-SUZ12, thus following the same pattern as EPOP and JARID2.

We also sought to confirm recent findings that JAZF1-SUZ12 exhibited an ectopic interaction with NuA4/TIP60 (Piunti et al., 2019). IP for JAZF1-SUZ12 or JAZF1 co-precipitated the NuA4 subunit TRRAP, but this was not the case for IP of SUZ12 or SUZ12Δ93 (Figure 1B). Therefore, fusion of SUZ12 with JAZF1 disrupts interaction with EPOP, JARID2, and PALI1 due to loss of the SUZ12 N terminus and induces interaction with TRRAP due to gain of the JAZF1 N terminus.

### JAZF1-SUZ12 exhibits reduced occupancy at PRC2 target genes and takes on a JAZF1-like binding profile during cell differentiation

**Figure 2:**
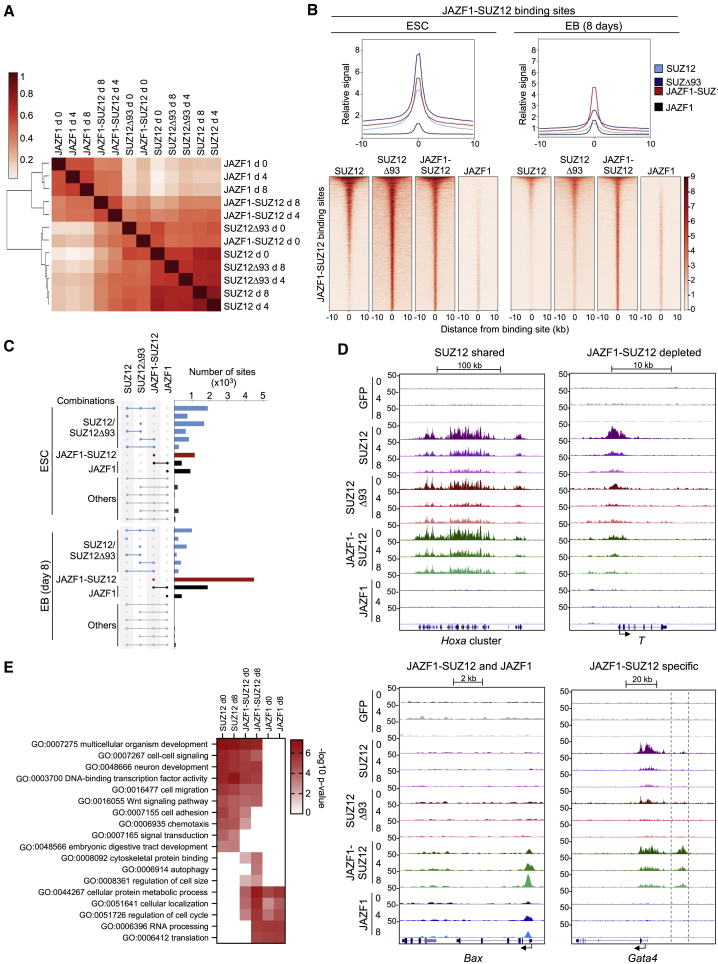
JAZF1-SUZ12 takes on a JAZF1-like binding profile during cell differentiation (A) Hierarchical clustering showing the relationship between the patterns of SUZ12, SUZ12Δ93, JAZF1-SUZ12, and JAZF1 genome occupancy in ESCs and embryoid bodies (EBs) 4 and 8 days after induction of EB formation (averages of two independent ChIPs). Pearson correlations between samples are shown by color, according to the scale on the left. (B) Metagene plots (above) and heatmaps (below) showing SUZ12, SUZ12Δ93, JAZF1-SUZ12, and JAZF1 occupancy at sites bound by JAZF1-SUZ12 in ESCs (left, n = 4,812) or EBs (right, n = 8,165). In the heatmaps, occupancy (normalized reads) is indicated by color, according to the scale on the right. At each time point, sites are ordered by the average occupancy of all factors. (C) UpSet plots showing the number of sites bound by different combinations of proteins in ESCs and EBs. Binding combinations are grouped into sites bound by SUZ12 and/or SUZ12Δ93 (light blue), sites bound by JAZF1-SUZ12 alone (red), sites bound by JAZF1-SUZ12 and/or JAZF1 (black), and other combinations (gray). (D) Genome occupancy (normalized reads per 10-bp window) at example genes bound by SUZ12, SUZ12Δ93, and JAZF1-SUZ12 (“SUZ12 shared”; *Hoxa* cluster); SUZ12, but not JAZF1-SUZ12 (“JAZF1-SUZ12 depleted”; *T*); JAZF1-SUZ12 and JAZF1, but not SUZ12 (*Bax*); and JAZF1-SUZ12 specific (dashed box upstream of *Gata4*) at 0, 4, and 8 days. (E) Heatmap showing enrichment (−log_10_ p value) of representative GO terms within the sets of genes occupied by SUZ12, JAZF1-SUZ12, or JAZF1 in ESCs (day 0) or EBs (day 8). See also Figure S2 and Table S1.

We next determined how fusion to JAZF1 affected PRC2 genome occupancy by performing calibrated chromatin immunoprecipitation sequencing (cChIP-seq) for FLAG-tagged GFP, SUZ12, SUZ12Δ93, JAZF1-SUZ12, and JAZF1 in ESC and at 4 and 8 days after induction of embryoid body (EB) formation. We first identified binding sites for each protein at each time point and used hierarchical clustering to determine how these were related (Figure 2A). SUZ12 and JAZF1 samples clustered separately, demonstrating they had distinct binding profiles. SUZ12Δ93 samples clustered together with SUZ12, indicating that loss of SUZ12 N terminus did not completely disrupt the pattern of PRC2 genome occupancy. Strikingly, the JAZF1-SUZ12 binding profile resembled SUZ12 and SUZ12Δ93 in ESC but was closer to JAZF1 in EBs. This shift of JAZF1-SUZ12 toward a JAZF1 binding profile was also apparent by comparing SUZ12 and JAZF1 occupancy at JAZF1-SUZ12 binding sites in ESCs versus EBs (Figure 2B). Furthermore, in contrast to SUZ12 and SUZ12Δ93, which tended to be depleted from their target genes during differentiation, JAZF1-SUZ12 was retained (Figure S2A).

To gain a more detailed picture of how fusion to JAZF1 affected SUZ12 genome occupancy, we quantified the number of binding sites shared between the different factors in ESCs and EBs (Figures 2C and S2B). Consistent with the hierarchical clustering results, this revealed that JAZF1-SUZ12 occupied a far greater proportion of JAZF1 binding sites in day 8 EBs (82%) than it did in ESCs (39%). JAZF1 target genes occupied by JAZF1-SUZ12 in this way included *Bax* and *Cbx3* (Figures 2D and S2C).

We next examined the effect of JAZF1 fusion on SUZ12 occupancy at its canonical binding sites. We found that around half (54%) of sites occupied by SUZ12 in ESCs were also occupied by SUZ12Δ93 and JAZF1-SUZ12, for example, the *Hoxa* cluster and *Pax3* (Figures 2C, 2D, S2B, and S2C). Of the remaining SUZ12 target sites, just under half (45%) exhibited reduced occupancy by both SUZ12Δ93 and JAZF1-SUZ12, including *T* (Brachyury) and *Fgf5*, while a similar number (39%) exhibited reduced occupancy by JAZF1-SUZ12 alone (Figures 2C, 2D, S2B, and S2C). Regions from which SUZ12Δ93 and/or JAZF1-SUZ12 were depleted tended to be narrower than regions at which binding was maintained (Figure S2B).

JAZF1-SUZ12 additionally bound a large number of sites that were not shared with SUZ12 or JAZF1, including upstream of *Gata4* and *Hhex*, and the number of these ectopic binding sites increased during ESC differentiation (from 54% to 79% of JAZF1-SUZ12 binding sites; Figures 2C, 2D, S2B, and S2C). In ESC, a large proportion (41%) of these ectopic JAZF1-SUZ12 binding sites were also occupied by SUZ12Δ93, suggesting that they were caused by loss of the SUZ12 N terminus, whereas the vast majority of ectopic binding sites in EBs (94%) were unique to JAZF1-SUZ12 (Figures 2C and S2B). We conclude that loss of the SUZ12 N terminus reduces JAZF1-SUZ12 occupancy at a subset of PRC2 target genes that exhibit narrower regions of occupancy and that fusion to JAZF1 triggers SUZ12 recruitment to JAZF1 target sites and to additional ectopic sites, especially in differentiated cells.

To determine how the changes in JAZF1-SUZ12 chromatin occupancy might impact cell state, we identified functional terms enriched in the sets of genes occupied by SUZ12, JAZF1-SUZ12, or JAZF1 (Figure 2E; Table S1). We found Gene Ontology (GO) terms related to development and transcription factor activity were shared between the sets of genes occupied by SUZ12 and JAZF1-SUZ12, but not by genes occupied by JAZF1. In contrast, other functional gene classes targeted by SUZ12, for example, cell adhesion and signal transduction, were lost from the set of genes occupied by JAZF1-SUZ12, either in both ESC and EBs or only in EBs. Instead, JAZF1-SUZ12 target genes exhibited functions in common with genes bound by JAZF1, including terms related to cell metabolism, translation, and cell cycle, and also functions specific to JAZF1-SUZ12 target genes, such as cytoskeletal protein binding and regulation of cell size. Thus, fusion to JAZF1 alters SUZ12 chromatin occupancy, and this changes the functional classes of genes targeted by the protein.

### JAZF1-SUZ12 reduces H3K27me3 and increases H4Kac at PRC2 target genes

**Figure 3:**
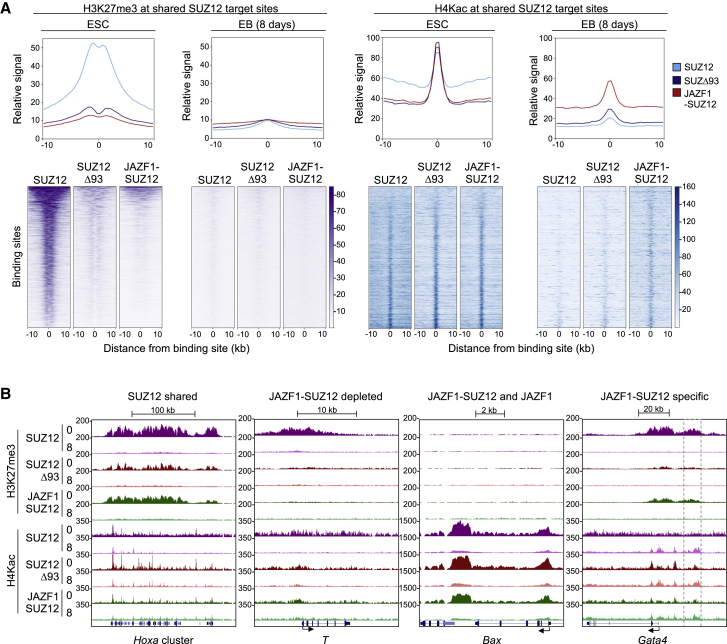
JAZF1-SUZ12 decreases H3K27me3 and increases H4Kac at PRC2 target genes (A) Metagene plots (above) and heatmaps (below) of H3K27me3 (left) and H4Kac (right) around sites shared by SUZ12, SUZ12Δ93, and JAZF1-SUZ12 in ESCs (n = 1,894) or EBs (n = 998) (averages of two independent ChIPs). In the heatmaps, occupancy (normalized reads) is indicated by color, according to the scales on the right. At each time point, sites are ordered by H3K27me3 in cells expressing SUZ12, from high to low. (B) H3K27me3 and H4Kac (reads per 10-bp window) at the regions shown in Figure 2D in ESCs expressing SUZ12, SUZ12Δ93, or JAZF1-SUZ12 at days 0 and 8 during differentiation. See also Figure S3.

We next used cChIP-seq to determine the effect of JAZF1-SUZ12 on H3K27me3, catalyzed by PRC2, and H4K5,8,12ac, catalyzed by NuA4/TIP60. To allow measurement of the effect of JAZF1-SUZ12 on H3K27me3 separately from its effect on PRC2 occupancy, we plotted H3K27me3 at target sites shared by SUZ12, SUZ12Δ93, and JAZF1-SUZ12 (Figure 3A). We found that ESCs expressing JAZF1-SUZ12 exhibited lower levels of H3K27me3 at PRC2 target sites than cells expressing WT SUZ12, even at broad regions of SUZ12 binding at which JAZF1-SUZ12 occupancy was maintained, such as the *Hoxa* cluster and *Pax3* (Figures 3B and S3A). At genes with narrower regions of SUZ12 occupancy from which JAZF1-SUZ12 binding was lost, such as *T* and *Fgf5*, H3K27me3 was almost completely absent in ESCs expressing JAZF1-SUZ12 (Figures 3B and S3A). This reduction in H3K27me3 was also observed in cells expressing SUZ12Δ93, demonstrating that it was caused by loss of the SUZ12 N terminus. Thus, fusion of JAZF1 to SUZ12 reduces H3K27me3, even at PRC2 target sites where the protein remains associated.

We also asked whether JAZF1-SUZ12 binding at non-PRC2 target sites, for example, at *Bax*, *Cbx3*, and *Hhex*, resulted in ectopic H3K27me3 (Figures 3B, S3A, and S3B). However, we found that H3K27me3 remained low at these sites in cells expressing JAZF1-SUZ12, consistent with the limited amounts of H3K27me3 deposited by the fusion protein at PRC2 target sites.

We next turned our attention to the effects of JAZF1-SUZ12 on H4Kac at PRC2 target sites. Expression of JAZF1-SUZ12 in ESCs increased H4Kac at broad regions of PRC2 binding but had less of an effect at narrower regions at which the modification was already present (Figures 3A, 3B, and S3A). Cells expressing SUZ12Δ93 also exhibited an increase in H4Kac at these sites, suggesting that much of this effect was due to loss of the repressive function of H3K27me3 rather than due to recruitment of NuA4/TIP60. However, in EBs, JAZF1-SUZ12 caused a marked increase in H4Kac at PRC2 target sites that was not observed for SUZ12Δ93, indicating this was due to JAZF1 fusion (Figures 3A, 3B, and S3A). We did not observe an increase in H4Kac at JAZF1-SUZ12 binding sites shared with JAZF1 (Figure S3B), likely because of endogenous NuA4/TIP60 activity at these sites. We conclude that, in addition to reducing H3K27me3, JAZF1-SUZ12 increased H4Kac at PRC2 target genes, especially in differentiated cells.

### JAZF1-SUZ12 disrupts gene expression during ESC differentiation

**Figure 4:**
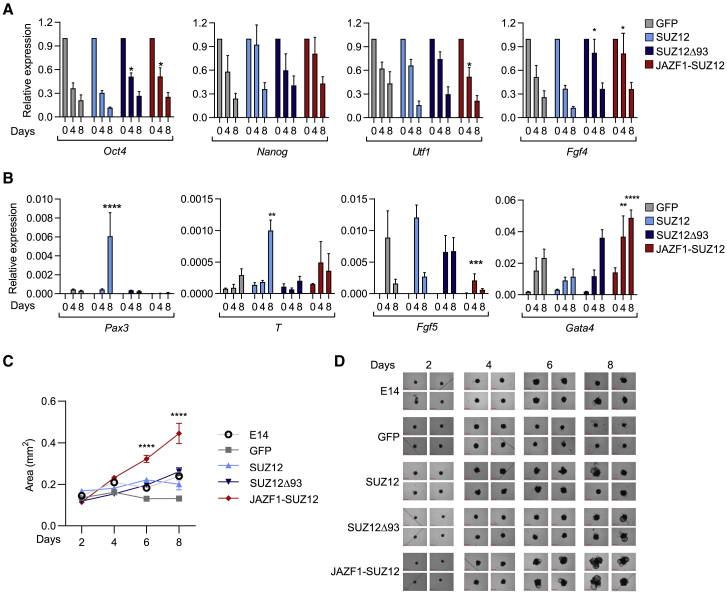
JAZF1-SUZ12 disrupts gene expression during cell differentiation (A) Expression of *Oct4*, *Nanog*, *Utf1*, and *Fgf4* (relative to *Gapdh* and day 0) at 0, 4, and 8 days after initiation of EB formation in *Suz12*^GT/GT^ ESCs expressing GFP, SUZ12, SUZ12Δ93, or JAZF1-SUZ12 (mean and SD of four independent EB inductions; ^∗^p < 0.05; two-way ANOVA). (B) Expression of *Pax3*, *T*, *Fgf5*, and *Gata4* (relative to *Gapdh*; other details as for A). (C) Size of EBs formed by WT and *Suz12*^GT/GT^ ESCs expressing GFP, SUZ12, SUZ12Δ93, or JAZF1-SUZ12 at days 2, 4, 6, and 8 (mean and SD; n ≥ 55 EBs; ^∗∗∗∗^p < 0.0001; two-way ANOVA). (D) Representative images of EBs from the time course described in (C). Scale bars represent 0.65 mm.

We considered that changes in PRC2 genome occupancy and histone modification induced by JAZF1-SUZ12 may be associated with alterations in gene expression during ESC differentiation. To address this, we harvested RNA from *Suz12*^GT/GT^ ESCs stably expressing GFP, SUZ12, SUZ12Δ93, or JAZF1-SUZ12 before and 4 and 8 days after induction of EB formation. First, examining pluripotency genes, we found that *Oct4*, *Fgf4*, *Nanog*, and *Utf1* were repressed during EB formation to a similar extent in all of the cell lines, although *Oct4* and *Fgf4* repression was delayed in cells containing SUZ12Δ93 or JAZF1-SUZ12 (p < 0.05; two-way ANOVA; Figure 4A).

Next, assessing the effect of JAZF1-SUZ12 on gene induction during EB formation, we found that JAZF1-SUZ12 disrupted normal patterns of gene induction but in different ways for different genes (Figure 4B). Unlike cells expressing SUZ12, cells expressing JAZF1-SUZ12 did not induce *Pax3* and also exhibited reduced induction of *Fgf5* and *T*. In contrast, *Gata4* was induced more strongly during differentiation of cells expressing JAZF1-SUZ12. SUZ12Δ93 had similar effects to JAZF1-SUZ12 on *Pax3* and *T* induction, indicating these changes were caused by loss of the SUZ12 N terminus, whereas the effects of JAZF1-SUZ12 on *Fgf5* and *Gata4* were distinct to those of SUZ12Δ93, indicating these changes were caused by fusion to JAZF1.

We next considered whether these alterations in gene induction in cells expressing JAZF1-SUZ12 were associated with changes in EB formation. We found that all cell lines formed EBs, and except for cells expressing GFP, these EBs increased in size between days 2 and 8, indicating some rescue of PRC2 function (Figures 4C and 4D). Strikingly, EBs formed by cells expressing JAZF1-SUZ12 were significantly larger than EBs formed by WT ESCs or *Suz12*^GT/GT^ ESCs expressing the other constructs (p < 0.0001; two-way ANOVA). Furthermore, this increase in EB size was associated with the formation of multiple cystic cavities (Figure 4D), which is associated with expression of endoderm markers, such as *Gata4* (Fujikura et al., 2002; Kulinski et al., 2015), consistent with activation of this gene by JAZF1-SUZ12. We conclude that JAZF1-SUZ12 dysregulates gene expression and this disrupts cell differentiation.

### JAZF1-SUZ12 activates polycomb target genes and dysregulates decidualization in primary hEnSCs

**Figure 5:**
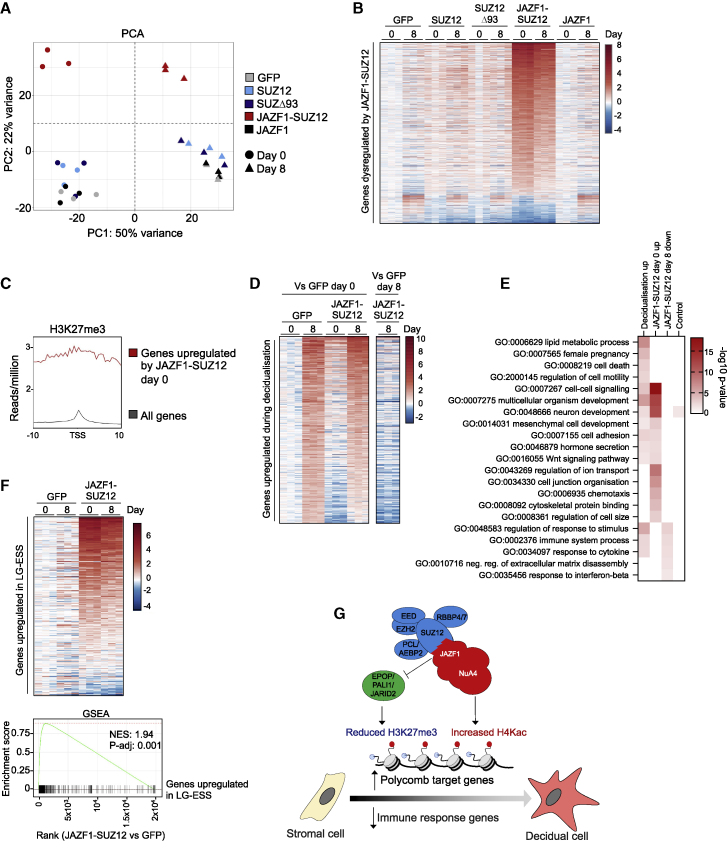
JAZF1-SUZ12 activates polycomb target genes and dysregulates decidualization in primary hEnSC (A) Principal component analysis of RNA-seq data from undifferentiated (day 0) and decidualized (day 8) hEnSCs from three donors expressing GFP, SUZ12, SUZ12Δ93, JAZF1-SUZ12, or JAZF1. (B) Genes (n = 909) that exhibit significant changes in expression in hEnSCs containing JAZF1-SUZ12 at day 0 or 8. The colors indicate log2 fold change in expression of these genes in hEnSCs from three donors containing GFP, SUZ12, SUZ12Δ93, JAZF1-SUZ12, or JAZF1 relative to the average expression in hEnSCs containing GFP at day 0, according to the scale on the right. (C) Metagene plot showing average H3K27me3 occupancy in hEnSCs around the transcription start site (TSS) of genes upregulated by JAZF1-SUZ12 (red) versus all genes (gray). (D) Genes (n = 453) significantly upregulated during decidualization of hEnSCs expressing GFP. Left: log2 fold change in expression of these genes in hEnSCs containing GFP or JAZF1-SUZ12 at days 0 and 8 relative to the average expression in hEnSCs containing GFP at day 0. Right: log2 fold change in expression of the same genes in hEnSCs containing JAZF1-SUZ12 at day 8 relative to hEnSCs containing GFP at day 8. (E) Enrichment (−log10 p value) of representative GO terms in sets of genes upregulated during decidualization of cells containing GFP, upregulated by JAZF1-SUZ12 in non-decidualized cells (day 0), downregulated by JAZF1-SUZ12 in decidualized cells (day 8), or a control set of genes that do not change in expression. (F) Top: expression of genes (n = 310) previously identified as upregulated in LG-ESS in hEnSCs containing GFP or JAZF1-SUZ12 relative to the average expression in hEnSCs containing GFP. Bottom: gene set enrichment analysis (GSEA) plotting the position of genes upregulated in LG-ESS in the set of genes ranked by change in expression in cells containing JAZF1-SUZ12 versus GFP. NES, normalized enrichment score. (G) Model. Loss of the SUZ12 N terminus prevents interaction of JAZF1-SUZ12 with EPOP, PALI1, and JARID2, decreasing PRC2 occupancy and reducing H3K27me3 at polycomb target genes. Fusion to JAZF1 induces association with NuA4/TIP60 and increases H4Kac at these sites. These changes are mirrored by ectopic activation of polycomb target genes in hEnSCs, inducing part of the decidualization gene expression program that lacks activation of immune response genes associated with clearance of senescent decidual cells from the endometrium. See also Figure S4 and Table S2.

Given that JAZF1-SUZ12 altered gene expression in ESCs, we asked whether this effect was also evident in the cell type in which the fusion event occurs, hEnSC. To assess this, we stably transduced primary hEnSCs with GFP, SUZ12, SUZ12Δ93, JAZF1-SUZ12, or JAZF1 (Figure S4A); harvested cells before and 8 days after induction of decidualization (Figure S4B); and performed RNA-seq. We found that SUZ12, SUZ12Δ93, and JAZF1 transgenes had little effect on the hEnSC gene expression program (Figures 5A and S4C). In contrast, JAZF1-SUZ12 caused large-scale changes to the hEnSC transcriptome, primarily in the form of gene upregulation (Figures 5B and S4C; Table S2).

Considering that JAZF1-SUZ12 triggered deposition of H4Kac at polycomb target genes in ESC, we hypothesized that the genes upregulated by JAZF1-SUZ12 were polycomb targets. To test this, we profiled H3K27me3 in hEnSCs by ChIP-seq and plotted the average level of this histone modification at genes upregulated by JAZF1-SUZ12 compared with other genes (Figure 5C). We found that genes upregulated by JAZF1-SUZ12 exhibited high levels of H3K27me3. There was also a significant overlap between the set of genes associated with H3K27me3 and the set of genes upregulated by JAZF1-SUZ12 (p = 3.2 × 10^−16^; hypergeometric; Figure S4D).

We next compared the gene expression changes caused by JAZF1-SUZ12 with those induced by decidualization. We discovered two striking relationships. Firstly, a significant proportion of genes upregulated during decidualization were also upregulated by JAZF1-SUZ12 in non-decidualized cells (p = 1.6 × 10^−18^; hypergeometric; Figures 5D and S4E), including *GREB1* (Camden et al., 2017; Katoh et al., 2018), *LGR5*, and *HES6* (Garcia-Alonso et al., 2021). For a second set of genes normally upregulated during decidualization, JAZF1-SUZ12 blocked this upregulation (p = 1.5 × 10^−16^; hypergeometric; Figures 5D and S4E). Thus, JAZF1-SUZ12 ectopically activated a portion of the decidualization gene expression program while repressing other aspects of the program.

To gain insight into these two sets of JAZF1-SUZ12-regulated genes, we performed GO analysis (Figure 5E). Genes upregulated by JAZF1-SUZ12 and genes upregulated during decidualization were both enriched for functions in development and cell-cell signaling, including the Wnt signaling pathway, characteristic of PRC2 target genes (Figure 2E). Other GO terms, including female pregnancy and cell death, were only enriched in the set of genes upregulated during decidualization. JAZF1-SUZ12 also activated genes with functions not normally induced during decidualization, including cytoskeleton and chemotaxis. Next, examining the set of genes downregulated by JAZF1-SUZ12 in decidualized cells, we found that this was enriched for functions related to the immune response, including *CXCL1*, *FOXO1*, and *PLAUR*, which are associated with senescent decidual stromal cells and their clearance by the immune system (Amor et al., 2020; Brighton et al., 2017; Gellersen et al., 2013; Rawlings et al., 2021). Thus, these findings demonstrate that JAZF1-SUZ12 activates genes related to hEnSC decidualization while blocking upregulation of genes with functions in cell death and immune-mediated clearance.

LG-ESS exhibits elevated expression of genes that are targeted by PRC2 in ESC (Przybyl et al., 2018). We therefore considered that dysregulated gene expression in LG-ESS might be related to the gene expression changes induced by JAZF1-SUZ12 in hEnSC. We found a significant enrichment of genes previously identified as highly expressed in LG-ESS (Przybyl et al., 2018) in the set of genes upregulated by JAZF1-SUZ12 (p_adj_ = 0.001; gene set enrichment analysis; Figure 5F). Furthermore, there was a significant overlap between the genes highly expressed in LG-ESS and those upregulated by JAZF1-SUZ12 (p = 4.3 × 10^−110^; hypergeometric; Figure S4F). These data are consistent with a model in which JAZF1-SUZ12-mediated alterations in hEnSC gene expression contribute to LG-ESS tumorigenesis.

## Discussion

We have determined the effect of JAZF1-SUZ12 on PRC2 composition, genome occupancy, histone modification, gene expression, and cell differentiation and distinguished effects caused by loss of the SUZ12 N terminus from those caused by fusion to JAZF1. JAZF1-SUZ12 thus exhibits both loss and gain of function, which leads to dysregulation of gene expression and cell differentiation (Figure 5G).

Our results reveal that the lack of interaction of JAZF1-SUZ12 with EPOP, PALI1, and JARID2 is due to loss of the SUZ12 N terminus. This is reflected by cryoelectron microscopy (cryo-EM) reconstructions that show the SUZ12 ZnB (amino acids 76–110) and Zn (amino acids 420–500) regions come together to form a structure that interacts with JARID2. That SUZ12 interacts with PALI1 and EPOP through this same region is consistent with the competition between JARID2 and EPOP for SUZ12 binding (Chen et al., 2018), the mutually exclusive association of PALI1 and EPOP with PRC2 (Alekseyenko et al., 2014; Hauri et al., 2016), and the finding that PALI1 mimics the allosteric activation function of JARID2 (Zhang et al., 2021).

JAZF1-SUZ12 and SUZ12Δ93 displayed differences in genome occupancy and H3K27me3 activity compared with WT SUZ12. The effect of JARID2 (Healy et al., 2019; Højfeldt et al., 2019; Landeira et al., 2010; Oksuz et al., 2018), EPOP (Beringer et al., 2016), and PALI1 (Conway et al., 2018) deficiency on PRC2 occupancy or H3K27me3 has been measured previously, but the effect of simultaneous loss of all of these interactions provides a comprehensive view of the role of the SUZ12 ZnB/Zn region. Although JAZF1-SUZ12 and SUZ12Δ93 binding was mostly maintained at broad regions, such as the *Hox* loci, the proteins were reduced at narrower regions, demonstrating a particular requirement for the ZnB/Zn region for recruitment to these sites.

Independent of these changes in occupancy, sites with comparable levels of JAZF1-SUZ12 and SUZ12 exhibited reduced H3K27me3 in cells expressing JAZF1-SUZ12. This was also the case for cells expressing SUZ12Δ93, indicating that the reduction in catalytic activity is due to loss of interaction with JARID2, EPOP, and PALI1 rather than fusion to JAZF1. This is consistent with the role of JARID2 (Sanulli et al., 2015) and PALI1 (Zhang et al., 2021) in the allosteric activation of EZH2. JAZF1-SUZ12 increased H4Kac at polycomb target sites, and this was also observed in ESCs expressing SUZ12Δ93, indicating that loss of repressive H3K27me3 was the primary cause. However, in EBs, JAZF1-SUZ12 had a more marked effect than SUZ12Δ93 on H4Kac, indicating gain of function caused by NuA4/TIP60 recruitment.

The loss of H3K27me3 and gain in H4Kac at polycomb target genes driven by JAZF1-SUZ12 in ESCs was reflected by the upregulation of polycomb target genes by the fusion protein in hEnSCs. hEnSCs differentiate into either decidual cells or a subpopulation of senescent decidual cells, which drive a transient inflammatory response associated with receptivity to embryo implantation and which are then cleared from the endometrium by uterine natural killer (uNK) cells (Brighton et al., 2017). In the absence of implantation, a second inflammatory decidual response leads to recruitment and activation of leukocytes and tissue breakdown (Gellersen and Brosens, 2014). JAZF1-SUZ12 ectopically upregulated a portion of the decidualization gene expression program, including genes with functions in cell adhesion and cell junction organization, which are characteristic of decidual cells. In contrast, JAZF1-SUZ12 did not upregulate other aspects of the decidualization gene expression program, including genes involved in cell death, and JAZF-SUZ12 suppressed the upregulation of genes associated with senescence and clearance by immune cells. The upregulation of genes with functions in cell adhesion coupled with the downregulation of genes associated with immune clearance suggests that JAZF1-SUZ12 may promote retention of cells in the endometrium (Figure 5G). That genes activated by JAZF1-SUZ12 in hEnSCs also exhibit elevated expression in LG-ESS suggests that the dysregulated form of decidualization driven by JAZF1-SUZ12 contributes to tumorigenesis.

### Limitations of the study

We found that JAZF1-SUZ12 exhibited a similar binding profile to WT SUZ12 in ESC but takes on a binding profile more similar to that of JAZF1 as cells differentiate. Further studies will be necessary to determine the cause of this shift in binding profile, but it may reflect changes in the expression of PRC2 and/or NuA4 subunits during ESC differentiation. Further work will also be necessary to determine whether JAZF1-SUZ12 increases other NuA4-mediated chromatin modifications (H2A.Z incorporation and H2A/H2A.Z acetylation) at polycomb target genes. Measurements of PRC2 composition, genome occupancy, and histone modification were performed in ESCs rather than in hEnSCs, in which JAZF1-SUZ12 may have different effects, and further studies will be required to determine the effect of JAZF1-SUZ12 on endometrial stromal cell decidualization *in vivo*.

In summary, we have defined the changes in PRC2 composition, genome occupancy, and activity caused by the JAZF1-SUZ12 fusion protein and the effects of these alterations on gene expression and cell differentiation. These results provide insights into the regulation of PRC2 function and the role of JAZF1-SUZ12 in LG-ESS tumorigenesis, which will provide opportunities for the treatment of this disease.

## STAR★Methods

### Key resources table

**Table:** 

REAGENT or RESOURCE	SOURCE	IDENTIFIER
**Antibodies**		

FLAG (M2)	Sigma	Cat# F3165; RRID:AB_259529
HA (3F10)	Roche	Cat# 12013819001; RRID: AB_390917
V5	Abcam	Cat# ab15828; RRID: AB_443253
FLAG-HRP	Sigma	Cat# P1379
SUZ12	Santa Cruz	Cat# sc-46264; RRID: AB_2196857
EZH2	Cell Signaling Technology	Cat# 3147; RRID: AB_10694383
JARID2	Cell Signaling Technology	Cat# 13594; RRID: AB_2798269
AEBP2	Cell Signaling Technology	Cat# 14129; RRID: AB_2798398
PCL2	Proteintech	Cat# 16208-1-AP; RRID: AB_2147370
EED	A. Bracken	Cat# 05–1320; RRID: AB_1586999
EPOP	L. Di Croce	Beringer et al. (2016)
TRRAP	Abcam	Cat# ab73546; RRID:AB_10672042
Alpha-tubulin	Cell Signaling Technology	Cat# 2144; RRID: AB_2210548
Beta-actin	Cell Signaling Technology	Cat# 4967; RRID:AB_330288
Histone H3	Abcam	Cat# ab1791; RRID:AB_302613
H3K27me3	Abcam	Cat# ab192985; RRID: AB_2650559
H4 pan-acetyl (K5,K8,K12)	Thermofisher	Cat# PA5-40083; RRID: AB_2608361

**Biological samples**		

Endometrial biopsies for primary human endometrial stromal cells	University Hospitals Coventry and Warwickshire (UHCW) NHS Trust, Implantation Clinic Coventry	https://www.tommys.org/research/research-centres/tommys-national-reproductive-health-biobank

**Chemicals, peptides, and recombinant proteins**		

Estradiol	Sigma	Cat# E2758
Insulin	Santa Cruz	Cat# sc-360248
Benzonase	Sigma	Cat# E1014
Leukemia inhibitory factor (LIF)	Stemgent	Cat# 03-0011-100
Polybrene	Merck	Cat# TR-1003-G
Medroxyprogesterone acetate (MPA)	Sigma	Cat# M1629
8-Br-cAMP	Merck	Cat# B5386
Polyethylenimine (PEI)	Polysciences	Cat# 23966
DNase Turbo	Ambion	Cat# AM2238
Dynabeads protein G	Invitrogen	Cat# 10003D
Streptactin high-capacity resin	IBA Life Sciences	Cat# 2-1208-002
Avidin	IBA Life Sciences	Cat# 2-0204-015
TRIsure	Bioline	Cat# BIO-38033
RNaseA	Sigma	Cat# R6513
Effectene	Qiagen	Cat# 301425
Kapa Pure beads	Roche	Cat# KK8000
DNase Turbo	Ambion	Cat# AM2238

**Critical commercial assays**		

NEBNext Ultra II DNA library preparation kit	NEB	Cat# E7645
Clarity ECL	Biorad	Cat# 1705061
ImProm-II Reverse Transcription System	Promega	Cat# A3800
KAPA Stranded RNA-Seq Kit with RiboErase	Roche	Cat# KR1151
KAPA Universal Library Quantification kit	Roche	Cat# KK4824
QuantiTect SYBR Green PCR kit	Qiagen	Cat# 204143
2100 Bioanalyzer High Sensitivity DNA kit	Agilent	Cat# 5067–4626
Agilent RNA 6000 Pico kit	Agilent	Cat# 5067–1513
Qubit dsDNA HS assay kit	Thermofisher	Cat# Q32851
Qubit RNA High Sensitivity assay kit	Thermofisher	Cat# Q32855

**Deposited data**		

ChIP-seq data	This paper	GEO: GSE169658
RNA-seq data	This paper	GEO: GSE169658
Western Blot raw image files	This paper	Mendeley Data: https://doi.org/10.17632/52rbsgb6by.1

**Experimental models: Cell lines**		

E14 ESC	Hooper et al. (1987)	RRID:CVCL_C320
*Suz12*^GT/GT^ ESC	Pasini et al. (2007)	N/A
NIH3T3	B. Vanhaesebroeck	RRID:CVCL_0594
Lenti-X 293T	Takara Biosciences	RRID:CVCL_4401
hTERT immortalised fibroblasts	Scaffidi and Misteli (2011)	N/A
Drosophila S2 cells	I. Bjedov	RRID:CVCL_Z232

**Oligonucleotides**		

Primers for RT-qPCR, see Table S3 provided	This work	N/A

**Recombinant DNA**		

pCAG-GFP-FLAG	A. Fisher	N/A
pCAG-SUZ12-FLAG	This work	N/A
pCAG-SUZ12Δ93-FLAG	This work	N/A
pCAG-JAZF1-SUZ12-FLAG	This work	N/A
pCAG-JAZF1-FLAG	This work	N/A
pCAG-FS2-GFP	This work	N/A
pCAG-FS2-SUZ12	This work	N/A
pCAG-FS2-SUZ12Δ93	This work	N/A
pCAG-FS2-JAZF1-SUZ12	This work	N/A
pCAG-FS2-JAZF1	This work	N/A
pMY-GFP-FLAG-IRES-bls	This work	N/A
pMY-SUZ12-FLAG-IRES-bls	This work	N/A
pMY-SUZ12Δ93-FLAG-IRES-bls	This work	N/A
pMY-JAZF1-SUZ12-FLAG-IRES-bls	This work	N/A
pMY-JAZF1-FLAG-IRES-bls	This work	N/A
pCBA-SUZ12-HA	This work	N/A
pCBA-SUZ12Δ93-HA	This work	N/A
pCBA-JAZF1-SUZ12-HA	This work	N/A
pCBA-JAZF1-HA	This work	N/A
pCAG-FS2-AEBP2	Grijzenhout et al. (2016)	N/A
pCAG-FS2-EPOP	Grijzenhout et al. (2016)	N/A
pLenti-FLAG-HA-PALI1	Conway et al. (2018)	N/A
PCL3-V5	Hunkapiller et al. (2012)	N/A
pCMV-Gag-Pol	G. Towers	Cat# RV-111
pMDG	G. Towers	N/A

**Software and algorithms**		

Ensembl BioMart	Smedley et al. (2009)	RRID:SCR_010714
Graphpad Prism version 8.0	GraphPad	RRID:SCR_002798
ImageQUant TL software	GE Life Sciences	RRID:SCR_014246
ImageJ version 1.50i	National Institutes of Health	RRID:SCR_003070
bowtie2 version 2.1.0	Langmead and Salzberg (2012)	RRID:SCR_016368
Bedtools	Quinlan and Hall (2010)	RRID: SCR_006646
DiffBind	Ross-Innes et al. (2012)	RRID:SCR_012918
MACS2	Feng et al. (2012)	RRID: SCR_013291
UpSetR	Conway et al. (2017)	N/A
Trim Galore	Babraham Institute	RRID:SCR_011847
STAR version 2.7.3a	Dobin et al. (2013)	RRID:SCR_004463
DeepTools version 3.0.2	Ramírez et al. (2014)	RRID:SCR_016366
featureCounts version 5.25	Liao et al. (2014)	RRID:SCR_012919
DEseq2	Love et al. (2014)	RRID:SCR_015687
ggplot2	Wickham (2016)	RRID:SCR_014601
Pheatmap version 1.2	Kolde (2012)	RRID:SCR_016418
EnhancedVolcano	Blighe et al. (2019)	RRID:SCR_018931
g:Profiler2 v1.2	Raudvere et al. (2019)	RRID:SCR_018190
Fgsea	Korotkevich et al., (2021)	RRID:SCR_020938
R version 3.6.1	R Core Team (2019)	RRID:SCR_001905

### Resource availability

#### Lead contact

Further information and requests for resources and reagents should be directed to and will be fulfilled by the lead contact, Richard Jenner (r.jenner@ucl.ac.uk).

#### Materials availability

Plasmids generated in this study are available upon request to the lead contact, Richard Jenner (r.jenner@ucl.ac.uk).

### Experimental model and subject details

#### Cell culture

E14 ESC (male) and *Suz12*^GT/GT^ (female; gift from Diego Pasini) ESC were cultured on 0.1% gelatin coated dishes with KO-DMEM (ThermoFisher, 10829018), 10% FBS validated for mouse ESC culture (ThermoFisher, 16141079), 5% knockout serum replacement (ThermoFisher, 10828028), non-essential amino acids (ThermoFisher, 11140035), 2 mM L-glutamine (ThermoFisher, 25030–024), 50 μM 2-mercaptoethanol (ThermoFisher, 31350010), 100 U/mL penicillin-streptomycin (ThermoFisher, 15140–122), 1 mM sodium pyruvate (ThermoFisher, 11360039) and 1000 U/mL leukemia inhibitory factor (Stemgent, 03-0011-100). NIH 3T3cells (male; gift from Bart Vanhaesebroeck) and Lenti-X 293T (female; Takara Bio Europe) were cultured in high glucose DMEM (Thermofisher, 31966–047), 10% FBS (Thermofisher, 10270–106) and penicillin-streptomycin. Immortalized primary human fibroblasts (male; gift from Paola Scaffidi) expressing human telomere reverse transcriptase (hTERT) (Scaffidi and Misteli, 2011) were grown in MEM (Thermofisher, 11095–080) supplemented with 15% FBS, 100 U/mL penicillin streptomycin and 2 mM L-glutamine. Drosophila S2 cells (male; gift from Ivana Bjedov) were grown in Schneider’s Drosophila Medium (ThermoFisher, Cat.no. 21720–02) supplemented with 10% heat-inactivated FBS (ThermoFisher, 10500064) and 25 U/mL of penicillin-streptomycin. All cell lines were tested negative for mycoplasma (Lonza, LT07-701). Cell lines were not authenticated.

#### Embryoid body formation

ESC were differentiated into EBs as described previously (Brien et al., 2012). ESC were filtered using a 70 μm cell strainer, washed twice with LIF-free media and seeded as a single cell suspension on ultra-low attachment T75 flasks (Corning, 3814) at 2 × 10^6^ cells per flask. LIF-free media was changed every second day and EBs were harvested at days 4 and 8. For quantification of EB growth, 200 cells per well were seeded in 200 μL of LIF-free media in a 96-well ultra-low attachment plate (Costar, 7007) and media was changed every third day. Pictures of single wells were taken with a EVOS FL Auto Imaging System at days 2, 4, 6 and 8, and EB diameter was measured by ImageJ (National Institutes of Health) using a macro that used the 650 μm bar in each image as reference.

#### Transfection and generation of cell lines

pCAG constructs were transfected into *Suz12*^GT/GT^ ESC with Effectene (Qiagene, 301425), following the manufacturer’s protocol, cells selected with 2 μg/mL puromycin and stable cell lines expanded from single cell colonies. NIH 3T3cells were transfected with 1 μg/mL plasmid with polyethyleneimine (3 μg/mL; Polysciences, 23966). Media was changed the next day and cells harvested 48 h later. Retroviruses were produced by transfection of lenti-X 293T cells with 1.5 μg of pMY-IRES-bls constructs, 1 μg of pCMV-Gag-Pol and 1 μg of pMDG (gifts from Greg Towers) using Fugene-HD (Promega, E2311). Virus was collected from 4 × 10 cm plates 48, 72 and 96 h after transfection and concentrated at 17,000 x g for 2 h at 4°C. The viral pellet was resuspended in 1 mL FBS-free DMEM/F12 and then supplemented with 10% DCC FBS prior to freezing at −80°C. To make the FLAG-SUZ12 spike-in control material for ChIP-seq, hTERT cells were transduced with 1.5 mL of concentrated pMY-SUZ12-FLAG-IRES-bls virus in the presence of 8 μg/mL polybrene (Merck, TR-1003-G). Cells were then spinoculated at 500 x g for 1 h at RT and incubated for 12 h. Cells were washed with fresh media and, after 3 days, selected with 5 μg/mL blasticidin.

#### hEnSC culture and transduction

The collection of endometrial biopsies was approved by the NHS National Research Ethics - Hammersmith and Queen Charlotte’s & Chelsea Research Ethics Committee (REC reference: 1997/5065) and Tommy’s National Reproductive Health Biobank (REC reference: 18/WA/0356). Samples were obtained using a Wallach Endocell sampler 5 to 10 days after the pre-ovulatory luteinizing hormone (LH) surge. hEnSC (female) were harvested as previously described (Barros et al., 2016). Endometrial biopsies were subjected to enzymatic digestion using 500 μg/mL collagenase type Ia (Sigma-Aldrich) and 100 μg/mL DNase I (Lorne Laboratories Ltd) for 1 h at 37°C. Digested tissue was filtered through a 40 μm cell strainer to remove glandular cell clumps, and the flow-through collected and cultured in DMEM/F12 (Thermo Scientific, 31330038) containing 10% dextran-coated charcoal-treated fetal bovine serum (DCC-FBS), 1 × antibiotic-antimycotic mix (ThermoFisher, 15240062), 10 μM L-glutamine, 1 nM estradiol (Sigma, E2758) and 2 μg/mL insulin (Santa Cruz, sc-360248). Cells were reseeded at a 1:2 or 1:3 ratio when confluence was reached. All experiments were carried out before reaching the 10^th^ passage.

hEnSC were seeded at a confluency of 10^5^ cells/well in 6 well plates and, two days later, 1.5 mL of concentrated retrovirus was added to each well in the presence of 8 μg/mL polybrene (Merck, TR-1003-G). Cells were spinoculated at 500 x g for 1 h at RT and incubated for 12 h. Cells were then washed with fresh media and, after 3 days, selected with 5 μg/mL blasticidin until they reached near confluency and no cells were present in the non-transduced control plate. Cells were reseeded and, at confluency two days later, decidualized for up to 8 days in dextran coated charcoal stripped and heat inactivated media with 2% DCC FBS supplemented with antibiotic antimycotic solution, L-glutamine, 50 μM 8-Bromoadenosine 3′, 5′ cyclic mono-phosphate (8-Br-cAMP, Merck B5386) and 1 μM medroxyprogesterone acetate (MPA, Sigma M1629), with the media changed every second day. Samples were taken at 0 and 8 days during decidualization.

### Method details

Experiments were not performed blinded. Samples were not randomised.

#### Cloning

Human SUZ12 and SUZ12Δ93 were PCR amplified from cDNA. JAZF1 was PCR amplified from IMAGE clone 4814463. To generate JAZF1-SUZ12, nucleotides encoding amino acids 1–128 of JAZF1 and amino acids 94–739 of SUZ12 were PCR amplified and joined in-frame using synonymous *AccIII* sites incorporated into the PCR primers. The constructs were cloned into pCBA-HA and into pCAG-GFP-2xFLAG (gift from Amanda Fisher). N-terminal SUZ12 deletions used for CLIP experiments were generated by PCR from the full-length SUZ12 construct. For tagging with Strep-tags, ORFs were PCR amplified from the pCAG plasmids (primers: 5′-TACTTCCAATCCATG and 5′-TATCCACCTTTACTG), C overhangs added with T4 DNA polymerase (M4211) and hybridised with *BaeI*-linearised pCAG-FS2-LIC (gift from Rob Klose) to which G overhangs had been added. To generate retroviral vectors, ORFs were excised from pCAG-2xFLAG with 5′*EcoRI* and 3′*NotI* and ligated into pMY-IRES-bls.

#### Co-immunoprecipitation and pull-down

Immunoprecipitations were performed as described (Conway et al., 2018), with minor modifications. 50 million cells per IP were suspended in 500 μL of high salt buffer (50 mM Tris-HCl, pH 7.2, 300 mM NaCl, 0.5% (v/v) NP-40, 1 mM EDTA pH7.4, Complete protease inhibitor (Roche, 11873580001) and 1 mM DTT) and sonicated for 3 × 10 s using a Bioruptor Pico (Diagenode). Cells were then rotated at 4°C for 20 min before the lysates were diluted with 500 μL of no salt buffer (50 mM Tris-HCl, pH 7.2, 0.5% (v/v) NP-40, 1 mM EDTA pH7.4, Complete protease inhibitor and freshly added 1 mM DTT). Afterward, lysates were clarified by centrifugation for 10 min at 17,000 x g at 4°C and 50 μL taken from the sample for use as input. The remainder of the lysate was incubated with 2 μg of anti-FLAG M2 (Sigma, F1804), SUZ12 (CST, 3737) or anti-V5 (Abcam, ab15828) antibody with 250 U/mL benzonase (Sigma, E1014) for 16 h 50 μL Protein G Dynabeads (Invitrogen, 10003D) per sample were washed 3 times with wash buffer (1:1 dilution of high salt: no salt buffer), resuspended in their initial volume, and incubated with the protein lysates for 2 h with rotation at 4°C. The flow-through was removed and immunocomplexed beads washed 5 times with 1 mL of wash buffer. Beads were resuspended in 100 μL of a 1:1 mix of 2X Laemmli buffer (4% SDS, 240 mM Tris pH 6.7, 2% beta-mercaptoethanol, 20% glycerol and 0.2% bromophenol blue) and 10 mM Tris HCl, while 50 μL of 2X Laemmli buffer was added to input samples and both heated at 95°C for 5 min. Finally, 10 μL of each IP and input sample were resolved by SDS-PAGE.

Strep-Tactin pull-down was carried out as for FLAG immunoprecipitation, with the following modifications. To block binding of any unspecific biotinylated proteins, 10 μg/mL of avidin (IBA, Cat No. 2-0204-015) was added to the extracts after cell lysis. Cell extracts were then incubated for 30 min at 4°C with rotation and centrifuged at 20,817 x g for 5 min at 4°C and the supernatant recovered. Supernatant was added to 10 μL of StrepTactin superflow high-capacity resin (IBA, Cat no. 2-1208-002) and incubated with rotation for 4 h at 4°C. Resin was then washed 5 times with wash buffer, each time pelleting the resin at 1000 x g for 5 min at 4°C. Protein was eluted by boiling the resin in Laemmli buffer.

#### Immunoblotting

Proteins were resolved alongside PageRuler (ThermoFisher, 26620) by SDS-PAGE using the Mini-PROTREAN Tetra Cell system (BioRad) in 200 mM glycine, 24 mM Tris base and 0.1% SDS. Proteins were transferred to 0.45 μM nitrocellulose membrane (GE Healthcare, 15269794) using a Mini Trans-Blot system (Biorad, 1610158) at 350 mA for 2 h. Membranes were blocked with 5% non-fat dried milk plus 0.1% Tween (Sigma, P1379) in TBS (TBST) for 1 h at RT. Proteins were detected with primary antibodies to FLAG M2 (Sigma, A8592), HA 3F10 (Roche, 12013819001), V5 (Abcam, ab15828), SUZ12 (Santa Cruz sc-46264), EZH2 (CST 3147), JARID2 (CST 13594), AEBP2 (CST 14129), PCL2 (Proteintech 16208-1-AP), EPOP (kind gift of L. Di Croce), TRRAP (Abcam, ab73546), FUS (Novus Biologicals 100–565), β-actin (CST 4967), alpha tubulin (CST 2144), H3K27me3 (Abcam ab192985), H4 pan-acetyl (Thermofisher, PA5-40083) and H3 (Abcam ab1791) and HRP-conjugated secondary antibodies (anti-mouse (Dako, P0447) or anti-rabbit (Dako, P0448). Proteins were visualised using the Clarity ECL Western Substrate (Biorad, 1705061) and detected using an ImageQuantLAS 4000 imager and ImageQuantTL software (GE Life Sciences). Contrast and brightness were altered in a linear fashion equally across the whole image.

#### Reverse transcription quantitative PCR (RT-qPCR)

RNA was purified using TRIsure (Bioline, BIO-38033), treated with DNaseTurbo (Ambion, AM2238) and reverse transcribed using the Im-Prom-II Reverse Transcription System (Promega, A3800) and random hexamer primers. Specific RNAs were quantified using the QuantiTect SYBR Green PCR Kit (Qiagen, 204145) and a QuantStudio 5 Real-Time PCR System (Thermofisher) with the primers listed in Table S3.

#### ChIP-sequencing (ChIP-Seq)

All ChIP-seq experiments were performed in duplicate, except for ChIP-seq for H3K27me3 in primary endometrial cells, for which only one experiment was performed. Cells were trypsinised, resuspended in PBS and then crosslinked by adding 1/10 volume of cross-linking solution (11% formaldehyde, 0.1 M NaCl, 1mM EDTA pH 8, 0.5 mM EGTA pH 8, 50 mM HEPES pH 8) for 15 min. Formaldehyde was quenched with 1.25 mM glycine. Cells were washed twice with ice-cold PBS, centrifuging at 290 x g at 4°C for 10 min each time, flash-frozen and stored at −80°C. Cells were thawed, resuspended in lysis buffer 1 (50 mM HEPES pH 7.5, 140 mM NaCl, 1 mM EDTA, 10% glycerol, 0.5% IGEPAL CA-630, 0.25% Triton X-100) and incubated at 4°C for 10 min with rocking. Cells were recovered by centrifugation at 290 x g for 10 min at 4°C and nuclei resuspended in the same volume of lysis buffer 2 (10 mM Tris pH 8, 200 mM NaCl, 1 mM EDTA, 0.5 mM EGTA, supplemented with 0.1 DTT and Complete protease inhibitor) and incubated at 4°C for 10 min with rocking. Nuclei were pelleted again at 290 x g for 10 min at 4°C and resuspended in 100 μL of lysis buffer 3 (10 mM Tris pH 8, 100 mM NaCl, 1 mM EDTA, 0.5mM EGTA, 0.1% sodium deoxycholate, 0.5% N-lauryl sarcosine, 0.2% SDS, supplemented with 0.1 DTT and Complete protease inhibitor) per 5 × 10^6^ cells. Cells were incubated for 30 min on ice and sonicated for 15 cycles of 30s on and 30s off using a Diagenode Bioruptor Pico. 2 × 10^7^ ESC or the EBs formed from 2 × 10 cm plates (4-day time point) or 1 × 10 cm plate (8 day time point) were used per ChIP. An aliquot of the sonicated chromatin was reverse crosslinked by heating at 65°C for 1 h, treated with 0.5 mg/mL RNaseA and 0.2 mg/mL of proteinase K (Ambion, AM2546). DNA was purified from this aliquot using KAPA Pure beads (Roche, KK8000), resuspended in 10 mM Tris pH 8 and quantified using the Qubit dsDNA HS Assay kit (Thermofisher, Q32851). Each ChIP was then performed with mouse chromatin equivalent to 45 μg of purified DNA. For ChIPs for FLAG-tagged proteins, sonicated human chromatin (extracted from hTERT cells stably expressing FLAG-SUZ12) equivalent to 5 μg of purified DNA was added as a calibration control. For histone modifications ChIPs, *Drosophila* chromatin (extracted from S2 cells) equivalent to 5 μg of purified DNA was added. The lysates were topped up to 900 μL per IP, 100 μL Triton X-100 10% solution added, and insoluble material removed by centrifugation at 17,000 x g for 20 min at 4°C. 2% of the lysate was stored at −20°C to be used as input and the remaining lysate was incubated overnight at 4°C with 50 μL protein G Dynabeads preincubated for at least 4 h with 2.5 μg of anti-FLAG M2 (Sigma, A8592), H3K27me3 (Abcam, ab192985) or H4 pan-acetyl (Thermofisher, PA5-40083) antibody. Beads were washed six times with wash buffer (50 mM HEPES, 1 mM EDTA, 0.5 M LiCl, 0.7% sodium deoxycholate, 1% NP-40) and once with TE with 50 mM NaCl and bound complexes eluted in elution buffer (50 mM Tris, 10 mM EDTA, 1% SDS). Crosslinks were reversed by heating at 65°C for 8 h and DNA purified as before.

Sequencing libraries were generated from 1.2 ng of DNA using the NEBNext Ultra II DNA library preparation kit (NEB, E7645) with 9 cycles of PCR. Library quality and size distribution was assessed using the 2100 Bioanalyzer High Sensitivity DNA Kit (Agilent, 5067–4626) followed by qPCR quantification with the Kapa Library Quant Kit (Roche, KK4824). FLAG libraries were subjected to 75 bp single-end sequencing on an Illumina NextSeq 550 platform and histone modification libraries were subjected to 138 bp single-end sequencing on an Illumina NovaSeq platform.

#### RNA-seq

Total RNA was purified from hEnSC using TRIsure (Bioline, BIO-38033), treated with DNaseTurbo (Ambion, AM2238), quantified with a Qubit RNA High Sensitivity assay kit (Invitrogen, Q32855) and its quality assessed using the Agilent RNA 6000 Pico Kit (Agilent, 5067–1513). Libraries were prepared with 100 ng of total RNA using the KAPA Stranded RNA-Seq Kit with RiboErase (Roche, KR1151) following the manufacturer’s instructions and were sequenced on an Illumina Nextseq 2000 to generate 2 × 50 bp paired-end reads.

### Quantification and statistical analysis

Statistical tests used and n are stated upon first use in the Results text and in the figure legends. No methods were used to determine whether the data met assumptions of the statistical approaches.

#### ChIP-seq data analysis

hEnSC H3K27me3 ChIP-seq data were aligned to hg19, as described (Beltran et al., 2016). GFP, SUZ12, SUZ12Δ93, JAZF1-SUZ12 and JAZF1 ChIP-seq reads were aligned to a concatenated genome sequence of mouse (mm10) and human (hg19) using bowtie2 with the --very-sensitive option (Langmead and Salzberg, 2012). Uniquely mapped reads were extracted using samtools (Li et al., 2009) and were used for the downstream analysis. For each sample, endogenous (mouse) and exogenous (human) reads were segregated into two bam files using samtools.

Spike-in calibration was performed as previously described (Fursova et al., 2019) and down-sampled bam files were generated for the endogenous mouse data. The two replicates for the endogenous data were merged and peaks were called using MACS2 with the --broad option and --broad-cutoff = 0.0001 (Feng et al., 2012). Peaks overlapping mouse blacklist regions (Amemiya et al., 2019) or peaks called in any of the GFP control datasets were removed using bedTools (Quinlan and Hall, 2010). Genome coverage tracks were obtained using the MACS2 pileup function, which were then converted to bigwigs and visualized with the UCSC Genome Browser (Kent et al., 2002).

A combined peak-set was created from the merged peaks from all the factors at the three time-points using DiffBind in R with the default parameters (Ross-Innes et al., 2012). Correlations between the peaks in different samples were calculated with DiffBind using the Pearson correlation co-efficient. Peak overlaps were calculated with dba.overlap and UpSet plots generated with UpSetR (Conway et al., 2017). Metaplots and heatmaps were generated with computeMatrix and plotProfile/plotHeatmap functions from deepTools (Ramírez et al., 2014).

H3K27me3 and H4Kac ChIP-seq reads were aligned to a concatenated genome sequence of mouse (mm10) and *Drosophila* (BDGP5.25) using bowtie2 with the --very-sensitive option. Uniquely mapped reads were extracted and separated into endogenous (mouse) and exogenous (*Drosophila*) reads using samtools. The normalization factor for the mouse bams was calculated using the *Drosophila* spike-in as previously described (Orlando et al., 2014). The normalization factor was used to generate the mouse bigwigs using the bamCoverage function from deepTools.

The closest gene transcription start site (TSS; Ensembl v98) to each SUZ12, JAZF1-SUZ12 and JAZF1 MACS peak was identified with bedTools. Gene TSS within 1 kb of a peak were considered bound. Gene Ontology terms enriched in the sets of genes bound by each factor were identified using g:Profiler with the default settings (Raudvere et al., 2019).

#### RT-qPCR analysis

Measurements of relative gene expression during EB formation were performed in quadruplicate (4 independent ESC cultures). Mean and standard deviation were plotted and the significance of differences between samples estimated using two-way ANOVA with GraphPad Prism.

#### Embryoid body size analysis

Mean and standard deviation were plotted and the significance of differences between samples estimated using two-way ANOVA in GraphPad Prism (E14 n = 95, GFP n = 86, SUZ12 n = 86, SUZ12Δ93 n = 96, JAZF1-SUZ12 n = 55 EBs).

#### RNA-seq data analysis

RNA-seq data were processed using the Nextflow core RNA-seq pipeline with default settings (Ewels et al., 2020). In brief, reads were filtered to remove adaptors and low-quality bases using Trim Galore, aligned to GRCh38 using the STAR aligner (Dobin et al., 2013), and read counts generated using featureCounts (Liao et al., 2014). Differential gene expression analysis between different factors and days was conducted using *DEseq2* (Love et al., 2014) and gene expression levels across all samples calculated and normalised against GFP day 0 or day 8 using the regularized logarithm transformation and visualised using *pheatmaps*. Volcano plots were generated using shrinked log2 fold changes with apeglm estimation (Zhu et al., 2019) and drawn using *EnhancedVolcano* (Blighe et al., 2019). Genes that had a log2 fold change >1 or < −1 and with p_adj_ < 0.001 were selected for downstream analyses.

Weighed Venn diagrams to show overlaps between gene lists were drawn using *eulerr*. GSEA was performed using *fgsea* (Korotkevich et al., 2021) and a set of 310 genes upregulated in LG-ESS taken from (Przybyl et al., 2018). Hypergeometric tests were performed in R using phyper(q, m, n, k, lower.tail = FALSE), where q = overlap-1, m = group2, n = total–group2 and k = group1.

## Data Availability

• ChIP-seq data from ESC and RNA-seq data from hEnSC have been deposited at Gene Expression Omnibus (GEO) and are publicly available as of the date of publication. The accession numbers are listed in the key resources table. • Raw western blot images have been deposited at Mendeley Data and are publicly available as of the date of publication. The accession number is listed in the key resources table. • This paper does not report original code. • Any additional information required to reanalyze the data reported in this paper is available from the lead contact upon request.

## Supplemental information

Document S1. Figures S1–S4

Table S1. Gene Ontology categories enriched in the sets of genes occupied by SUZ12, JAZF1-SUZ12, and JAZF1 in ESC and EBs, related to Figure 2

Table S2. Genes differentially expressed in hEnSCs containing JAZF1-SUZ12 at day 0 and during decidualization, related to Figure 5

Table S3. Oligonucleotide sequences, related to STAR Methods

Document S2. Article plus supplemental information
