# Supplementary material for: JAZF1-SUZ12 dysregulates PRC2 function and gene expression during cell differentiation
Source: Cell Rep. 2022 May 31;39(9):110889. doi: 10.1016/j.celrep.2022.110889 (PMC9637993; doi:10.1016/j.celrep.2022.110889)
Supplement: Document S1. Figures S1–S4 [file mmc1.pdf]

Cell Reports, Volume *39*

Supplemental information

JAZF1-SUZ12 dysregulates PRC2 function and gene expression during cell differentiation

Manuel Tavares, Garima Khandelwal, Joanne Muter, Keijo Viiri, Manuel Beltran, Jan J. Brosens, and Richard G. Jenner

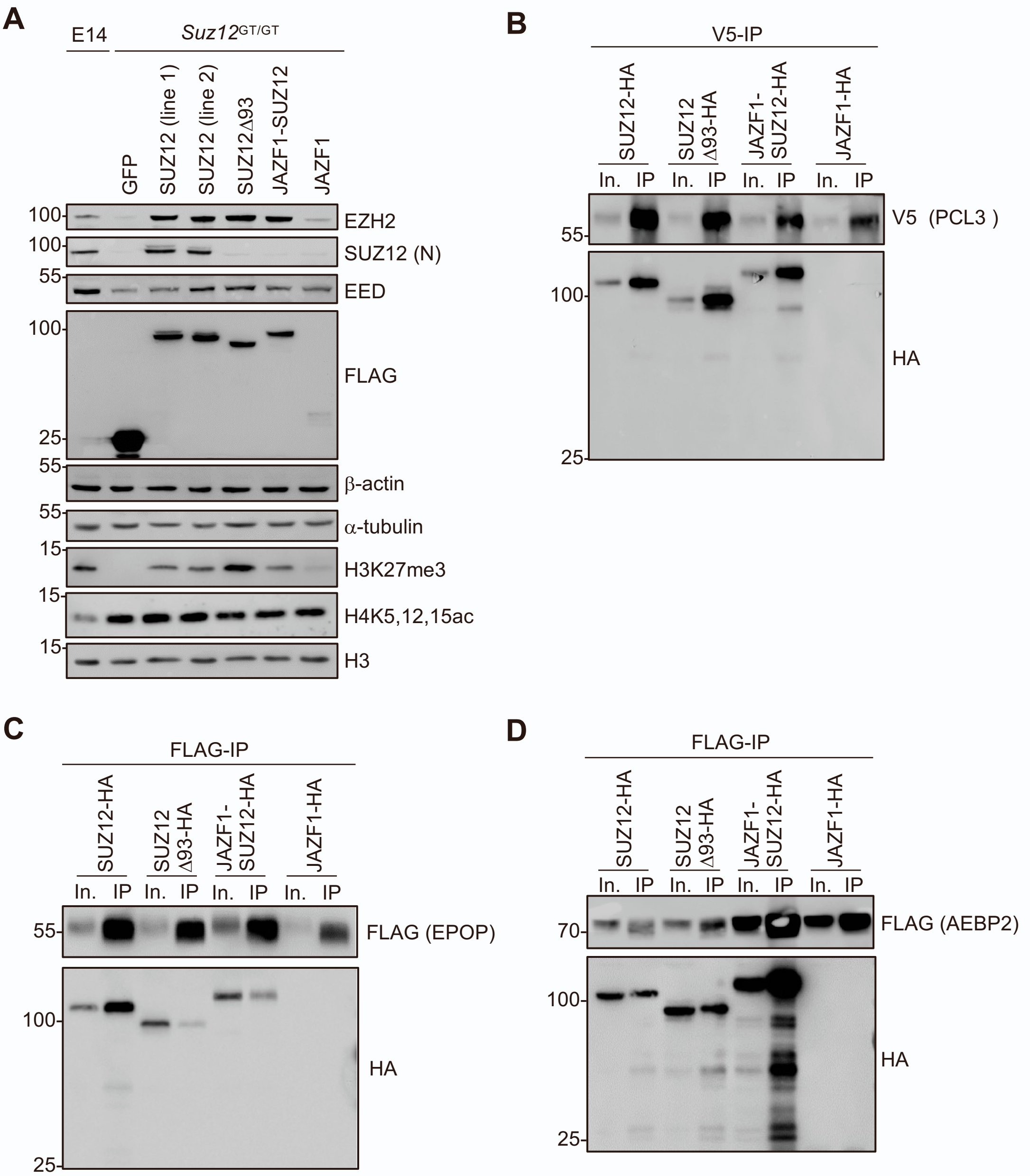

Figure S1. JAZF1-SUZ12 interacts with PCL3 and AEBP2 but shows reduced interaction with EPOP (related to Figure 1).

A. Levels of FLAG-tagged GFP, SUZ12 (2 independent cell lines), SUZ12Δ93, JAZF1-SUZ12, JAZF1 and H3K27me3 and H5K5,12,15ac in stable *Suz12*$^{GT/GT}$ cell lines in comparison to WT E14 cells.

B. Immunoblots for V5 and HA in input (In.) and SUZ12 IP fractions from NIH-3T3 cells co-transfected with V5-tagged PCL3 and HA-tagged SUZ12/JAZF1 constructs.

C. As B., except in cells transfected with FS2-EPOP and HA-tagged SUZ12/JAZF1 constructs.

D. As B., except in cells transfected with FS2-AEBP2 and HA-tagged SUZ12/JAZF1 constructs.

All images are representative of two independent IPs.

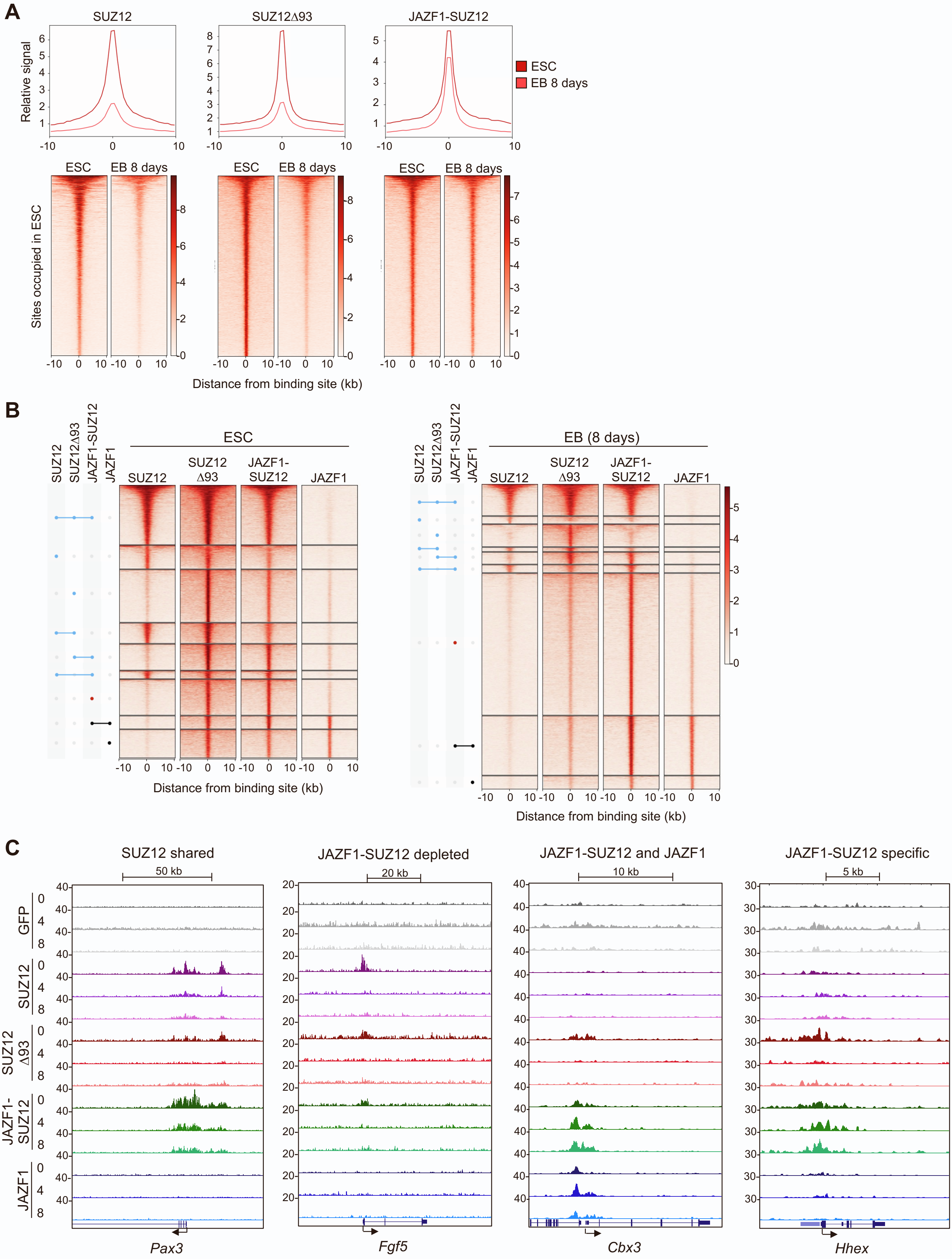

A
SUZ12
SUZ12Δ93
JAZF1-SUZ12
Relative signal
ESC
EB 8 days
Sites occupied in ESC
Distance from binding site (kb)
B
SUZ12
SUZ12Δ93
JAZF1-SUZ12
JAZF1
ESC
EB (8 days)
Distance from binding site (kb)
C
SUZ12 shared
JAZF1-SUZ12 depleted
JAZF1-SUZ12 and JAZF1
JAZF1-SUZ12 specific
50 kb
20 kb
10 kb
5 kb
GFP
SUZ12
SUZ12 Δ93
JAZF1-SUZ12
JAZF1
Pax3
Fgf5
Cbx3
Hhex

Figure S2. Differences in chromatin occupancy between JAZF1-SUZ12 and SUZ12 (related to Figure 2).

A. Metagene plots (above) and heatmaps (below) showing SUZ12, SUZ12Δ93 or JAZF1-SUZ12 occupancy in ESC and day 8 EBs at sites occupied by each factor in ESC (averages of two independent ChIPs). Occupancy (normalised reads) is indicated by color, according to the scales on the right. For each factor, binding sites are ordered by occupancy in ESC, from high to low.

B. Heatmaps showing SUZ12, SUZ12Δ93, JAZF1-SUZ12 or JAZF1 occupancy in ESC (left) and day 8 EBs (right) at sites bound by the various combinations of factors shown in Figure 2C. Whether a factor was bound to a site was determined using MACS ($q<0.0001$). Occupancy (normalised reads) is indicated by color, according to the scale on the right. For each factor and binding combination set, binding sites are ordered by occupancy from high to low. Evidence for binding can sometimes be observed for factors that were not called as bound by MACS at $q<0.0001$. But in these cases, the level of occupancy versus background is lower than for factors that were judged to be bound at these sites.

C. Further examples of the 4 major patterns of JAZF1-SUZ12 occupancy shown in Figure 2D**.** Left: shared SUZ12, SUZ12Δ93 and JAZF-SUZ12 binding at *Pax3.* Centre left: depletion of JAZF1-SUZ12 at *Fgf5.* Center right: Shared JAZF1-SUZ12 and JAZF1 occupancy at *Cbx3.* Right: JAZF1-SUZ12-specific binding at *Hhex* in day 4 and day 8 EBs.

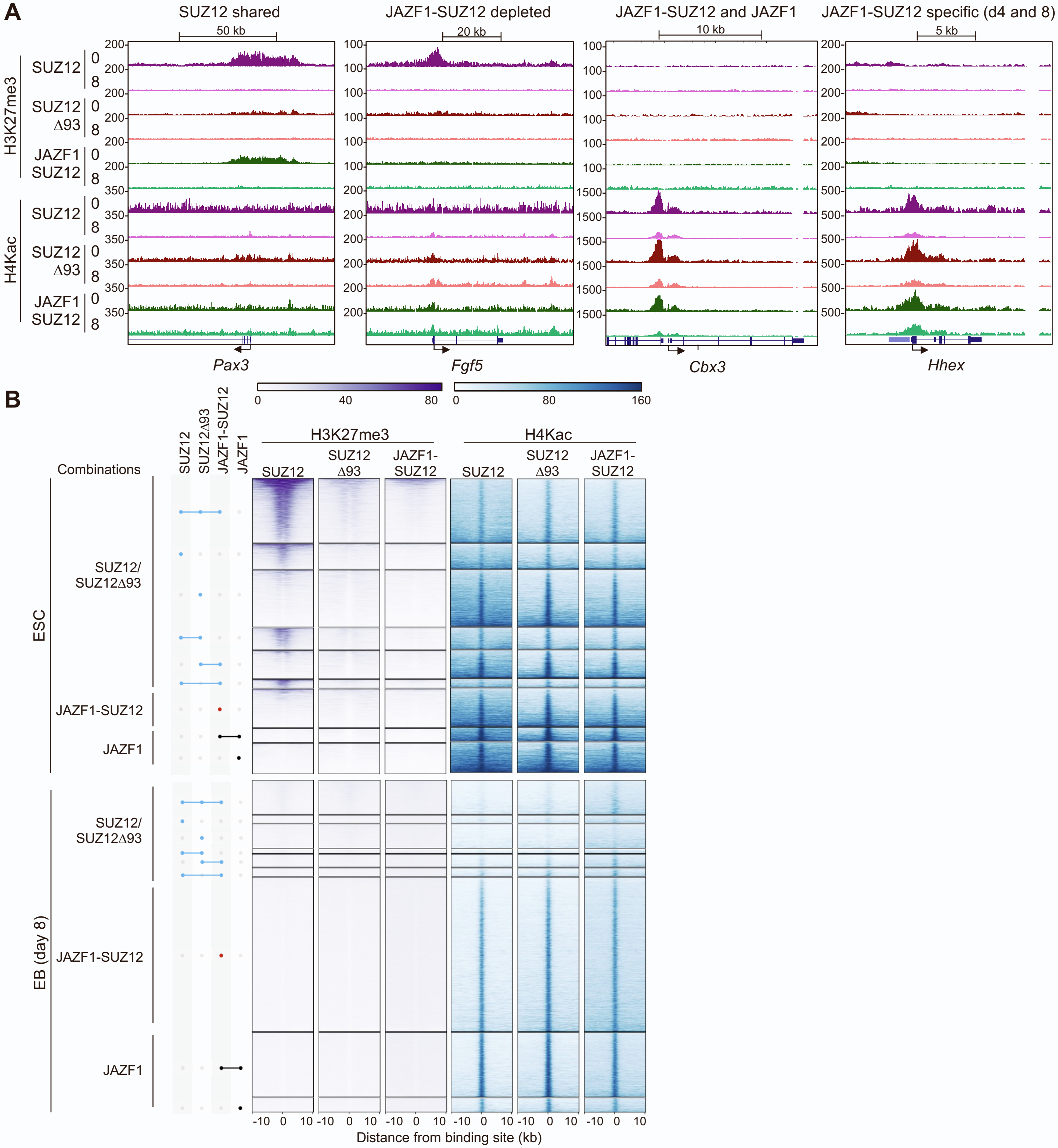

Figure S3. Changes in H3K27me3 and H4Kac in cells expressing JAZF1-SUZ12 (related to Figure 3).

A. H3K27me3 and H4Kac (reads per 10bp window) at the regions shown in Figure S2C in ESC expressing SUZ12, SUZ12Δ93 or JAZF1-SUZ12 at days 0 and 8 after initiation of differentiation into EBs.

B. Heatmaps of H3K27me3 (purple, left) and H4Kac (blue, right) in ESC (top) and in EB (bottom) at sites bound by the various combinations of factors shown in Figure 2C. Occupancy (normalised reads) is indicated by color, according to the scales above. For each binding combination, sites are ordered by H3K27me3 in cells expressing SUZ12, from high to low.

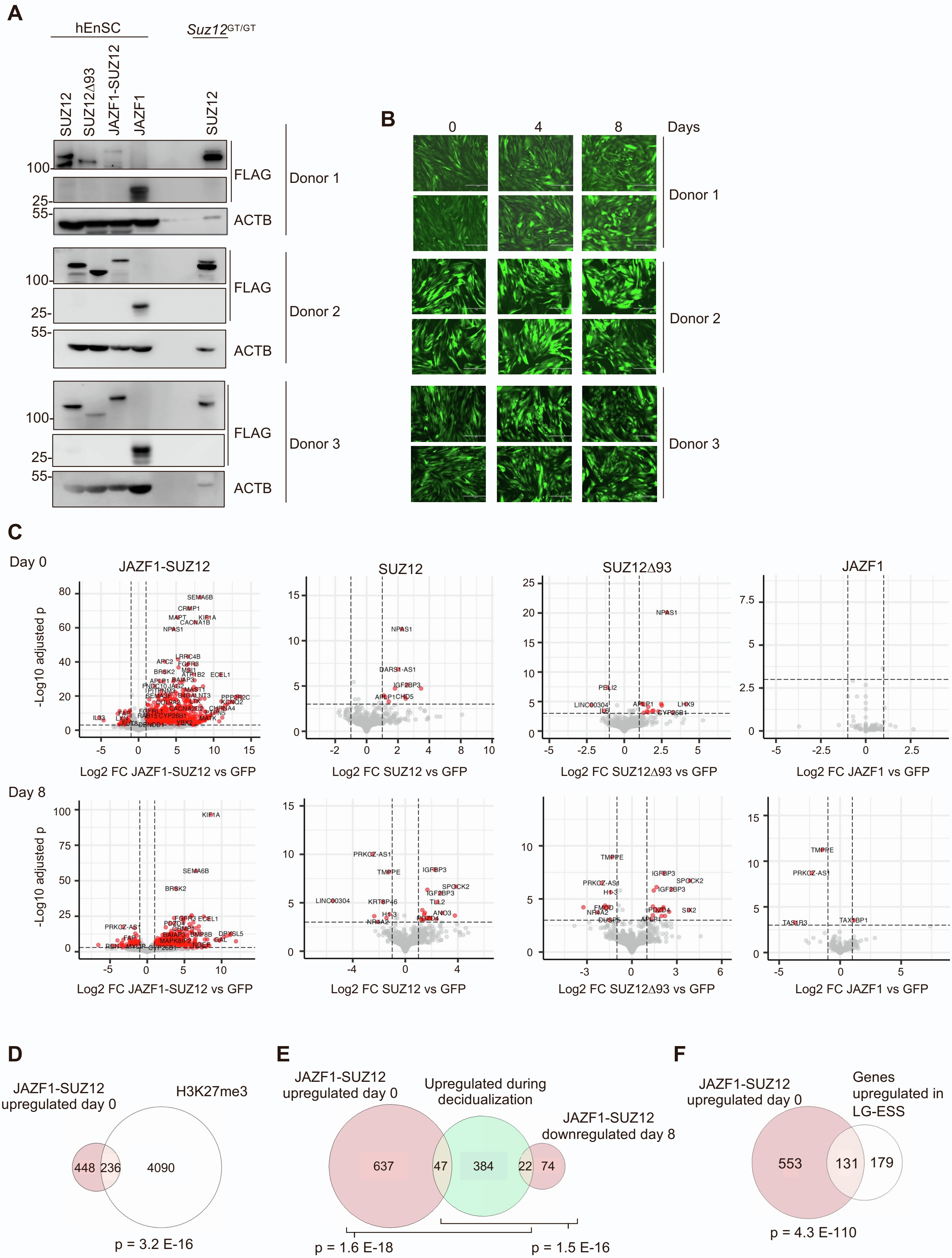
A
hEnSC
Suz12GT/GT
SUZ12
SUZ12Δ93
JAZF1-SUZ12
JAZF1
SUZ12
FLAG
ACTB
Donor 1
Donor 2
Donor 3
100-
25-
55-
B
0
4
8
Days
Donor 1
Donor 2
Donor 3
C
Day 0
JAZF1-SUZ12
SUZ12
SUZ12Δ93
JAZF1
-Log10 adjusted p
Log2 FC JAZF1-SUZ12 vs GFP
Log2 FC SUZ12 vs GFP
Log2 FC SUZ12Δ93 vs GFP
Log2 FC JAZF1 vs GFP
Day 8
SEMA6B
CRMP1
MAPT
KIF1A
CACNA1B
NPAS1
DARS1-AS1
IGF2BP3
PBLI2
LINC00304
APLP1
LHX9
CYP26B1
PRKCZ-AS1
TMPPE
SPOCK2
TAX1BP1
TAS1R3
D
JAZF1-SUZ12
upregulated day 0
H3K27me3
448
236
4090
p = 3.2 E-16
E
JAZF1-SUZ12
upregulated day 0
Upregulated during
decidualization
JAZF1-SUZ12
downregulated day 8
637
47
384
22
74
p = 1.6 E-18
p = 1.5 E-16
F
JAZF1-SUZ12
upregulated day 0
Genes
upregulated in
LG-ESS
553
131
179
p = 4.3 E-110

Figure S4. Effects of JAZF1-SUZ12 on gene expression in hEnSC (related to Figure 5).

A. Immunoblots for FLAG and ACTB in whole cell lysates of hEnSC stably expressing FLAG-tagged SUZ12, SUZ12Δ93, JAZF1-SUZ12 or JAZF1 in comparison to *Suz12*$^{GT/GT}$ ESC stably expressing FLAG-tagged SUZ12.

B. Representative images of hEnSC from 3 donors expressing GFP-FLAG at 0, 4 and 8 days after initiation of decidualization with cAMP and MPA showing gain of an epithelioid phenotype. The scale bars are 400 μm.

C. Volcano plots showing genes down or up-regulated at days 0 (above) and 8 (below) during decidualisation of hEnSC from 3 donors expressing JAZF1-SUZ12, SUZ12, SUZ12Δ93 or JAZF1 relative to cells expressing GFP. Limits (log2 fold change = |1| and adjusted p-value = 0.001) are shown as dashed lines. Genes with significant expression changes are shown as red points, with selected genes named; genes with non-significant changes are shown as gray points.

D. Venn diagram showing the overlap between the set of genes upregulated at day 0 in hEnSC expressing JAZF1-SUZ12 (red, n=684) with the set of genes with H3K27me3 peaks within 5 kb of their TSS in hEnSC (white, n=4326). Significance of the overlap (hypergeometric test) is shown below.

E. Venn diagram showing the overlap between the set of genes upregulated at day 0 (n=684) or downregulated at day 8 (n=96) by JAZF1-SUZ12 (red, left and right, respectively) and the set of genes upregulated during decidualisation (green, n=453). Significance of the overlap (hypergeometric test) is shown below each pairwise comparison.

F. Venn diagram showing the overlap between the set of genes that are upregulated at day 0 in hEnSC expressing JAZF1-SUZ12 (red, n=684) and the set of genes upregulated in LG-ESS reported by Przybyl et al., 2018 (white, n=310). Significance of the overlap (hypergeometric test) is shown below.
